# SpliceNet: recovering splicing isoform-specific differential gene networks from RNA-Seq data of normal and diseased samples

**DOI:** 10.1093/nar/gku577

**Published:** 2014-07-17

**Authors:** Hari Krishna Yalamanchili, Zhaoyuan Li, Panwen Wang, Maria P. Wong, Jianfeng Yao, Junwen Wang

**Affiliations:** 1 Department of Biochemistry, The University of Hong Kong, Hong Kong (SAR), China; 2 Shenzhen Institute of Research and Innovation, The University of Hong Kong, Shenzhen, China; 3 Department of Statistics & Actuarial Science, Faculty of Science, The University of Hong Kong, Hong Kong (SAR), China; 4 Department of Pathology, The University of Hong Kong, Hong Kong (SAR), China; 5 Centre for Genomic Sciences, L.K.S. Faculty of Medicine, The University of Hong Kong, Hong Kong (SAR), China

## Abstract

Conventionally, overall gene expressions from microarrays are used to infer gene networks, but it is challenging to account splicing isoforms. High-throughput RNA Sequencing has made splice variant profiling practical. However, its true merit in quantifying splicing isoforms and isoform-specific exon expressions is not well explored in inferring gene networks. This study demonstrates SpliceNet, a method to infer isoform-specific co-expression networks from exon-level RNA-Seq data, using large dimensional trace. It goes beyond differentially expressed genes and infers splicing isoform network changes between normal and diseased samples. It eases the sample size bottleneck; evaluations on simulated data and lung cancer-specific ERBB2 and MAPK signaling pathways, with varying number of samples, evince the merit in handling high exon to sample size ratio datasets. Inferred network rewiring of well established Bcl-x and EGFR centered networks from lung adenocarcinoma expression data is in good agreement with literature. Gene level evaluations demonstrate a substantial performance of SpliceNet over canonical correlation analysis, a method that is currently applied to exon level RNA-Seq data. SpliceNet can also be applied to exon array data. SpliceNet is distributed as an R package available at http://www.jjwanglab.org/SpliceNet.

## INTRODUCTION

Cancer is a complex biological phenomenon where the dynamic interplay between various tumor associated genes and their splice variants (isoforms) are curtailed in determining cell fate (1). With the progress in various graph theoretic techniques it is advantageous to map complex biological systems as networks/graphs (2). Network representation of such functional interactions provides an intuitive advantage in visualizing and in systematically understanding the cause and prognosis of various biological phenomena including cancer (3,4).

Traditionally, DNA microarrays are used to quantify gene expression patterns (5). Several studies demonstrated the merits of microarrays in discerning cancer and other biological phenomena (6,7). However, it is still challenging to account for the entire transcriptome using microarrays, especially in quantifying splice variations (8). Alternative splicing is the major factor that leads to functional diversity of proteins and various complications (1,9), almost half of the human genes undergo alternative splicing (10). Often different splice variants (isoforms) vary in their expression in different conditions, making them primary targets to explain biological anomalies (11). Splice variants are found to be associated with different cancers viz. spleen tyrosine kinase isoform-S (SkyS) (12) and human epidermal growth factor receptor (HER-2) (13) in breast cancer, B-cell lymphoma-extra large (Bcl-xL), Kruppel-like factor 6 (KLF6) and peroxisome proliferator-activated receptor gamma 1 (PPARγ1) in lung cancer (14) etc.

With the recent advances in next-generation sequencing, RNA Sequencing (RNA-Seq) is gaining popularity in accurately quantifying gene expression. RNA-Seq with its high sensitivity, low background noise and a larger range of coverage, is more robust when compared to traditional microarrays (15). In RNA-Seq experiments, RNA is firstly reverse transcribed and then sequenced. Sequences’ reads are then mapped to the reference genome. The gene expression is quantified according to the abundance of mapped cDNA. RNA-Seq offers a holistic picture of transcriptome by significantly enhancing gene expression analysis both qualitatively and quantitatively at multiple resolutions viz. spliced variants, post-transcriptional RNA editing, exon-level expression and allele-specific expression (15). In addition, RNA-Seq experiments can also reveal novel transcripts, non-coding RNA and other small RNAs that are not probed using microarrays. It is well recognized that splice variants along with other genomic variations are important cancer driving factors (16). The variations in non-coding genes and isoforms at exon-level can be efficiently captured by RNA-Seq (8). Profiling such variations in cancer patients using RNA-Seq experiments is a promising approach in identifying potential biomarkers for cancer prognosis, diagnosis and therapeutic targets.

Traditional gene network inference methods such as correlation or mutual information based methods, covariance selection, sparse graphical models and partial correlation methods are based on overall gene expressions (17). However, RNA-Seq data offer a significantly increased level of biological details (at base resolution) than just overall gene expressions. It is necessary to explore expression difference in genomic positions, exons and isoforms to identify potential cancer biomarkers and therapeutic targets. Recently Canonical Correlation Analysis (CCA) (18) is applied to RNA-Seq data to infer co-expression network using exon level expression data. Likelihood ratio test (LRT) can also be used to infer the multivariable (exon expression) dependency between two genes (19). However, the merit of RNA-Seq in quantifying splicing isoforms is not explored in inferring isoform-specific networks. Moreover, CCA and LRT are designed under the assumption that the number of dimensions (exons per gene) is small while the sample size tends to large. When the ratio of exons to sample size is not small enough the results from corresponding methods are not consistent. It may not be always practical to have sample size much larger than the number of dimensions (exons per gene); small number of available tumor and normal matched RNA-Seq samples support the argument.

It is also important to account for isoform-specific exon expressions, as an exon can be shared by multiple isoforms with different expressions. Unfortunately, none of the current methods consider isoform-specific exon expressions. In lieu of above, there is a strong need to develop efficient computational methods for RNA-Seq expression data analysis that can account isoform-specific exon expressions and are least affected by the exons to sample size ratio (20). This study proposes a novel method to address the challenges in investigating large multi-dimensional RNA-Seq data. To construct co-expression networks with isoform resolution, firstly expressions of isoforms/genes are abstracted as multivariate variables (matrices). Next, a novel method, large dimensional trace test (LDT), is employed to recover corresponding pairwise dependencies. In brief, a co-expression edge is inferred by accepting or rejecting the null hypothesis that is centered on the variance matrix of respective isoform expressions (exon-expression matrices). The proposed method hypothesizes an asymptotic distribution on the trace of variance matrix using large dimensional theory, which makes it more robust to the difference between number of exons and number of RNA-Seq samples.

The networks recovered by the proposed method perceive isoform co-expressions. This study goes beyond differentially expressed genes and comprehends diseases by inferring isoform network differences, and can be used in understanding the molecular mechanisms of cancer and other diseases (21). Furthermore, the method can also be applied to infer isoform mediated auto-regulatory relationships (22) by computing intra-genic isoform dependencies. An R package implementing the proposed approach for constructing isoform-specific co-expression networks from exon level RNA-Seq data, SpliceNet can be downloaded from our website http://www.jjwanglab.org/SpliceNet/. Although this study demonstrates the application of SpliceNet to cancer genomic data, it can be applied to any exon level RNA-Seq data or exon array data. A detailed explanation of the proposed approach is given in the ‘Materials and Methods’ section.

## MATERIALS AND METHODS

### Datasets

Exon-level (level 3) RNA-Seq data of lung, kidney and liver cancers are downloaded from TCGA data portal. In total 49 lung adenocarcinoma (LUAD), 45 lung squamous cell carcinoma (LUSC), 50 liver hepatocellular carcinoma (LIHC) and 72 kidney renal cell carcinoma (KIRC) matched samples are used in this study. An in-depth description of RNA-Seq data is published elsewhere (23). Cancer-specific ERBB2 and MAPPK signaling pathways are collected from KEGG database (24). Tissue-specific gene expression profiles and gene expression correlations are downloaded from TiGER database (25) and Ensembl's Human BodyMap project 2.0 (26) respectively. Gene symbol to Refseq ID mapping and their corresponding exon boundaries are obtained from UCSC genome browser (27).

### Constructing exon-expression matrix

**Figure 1.**
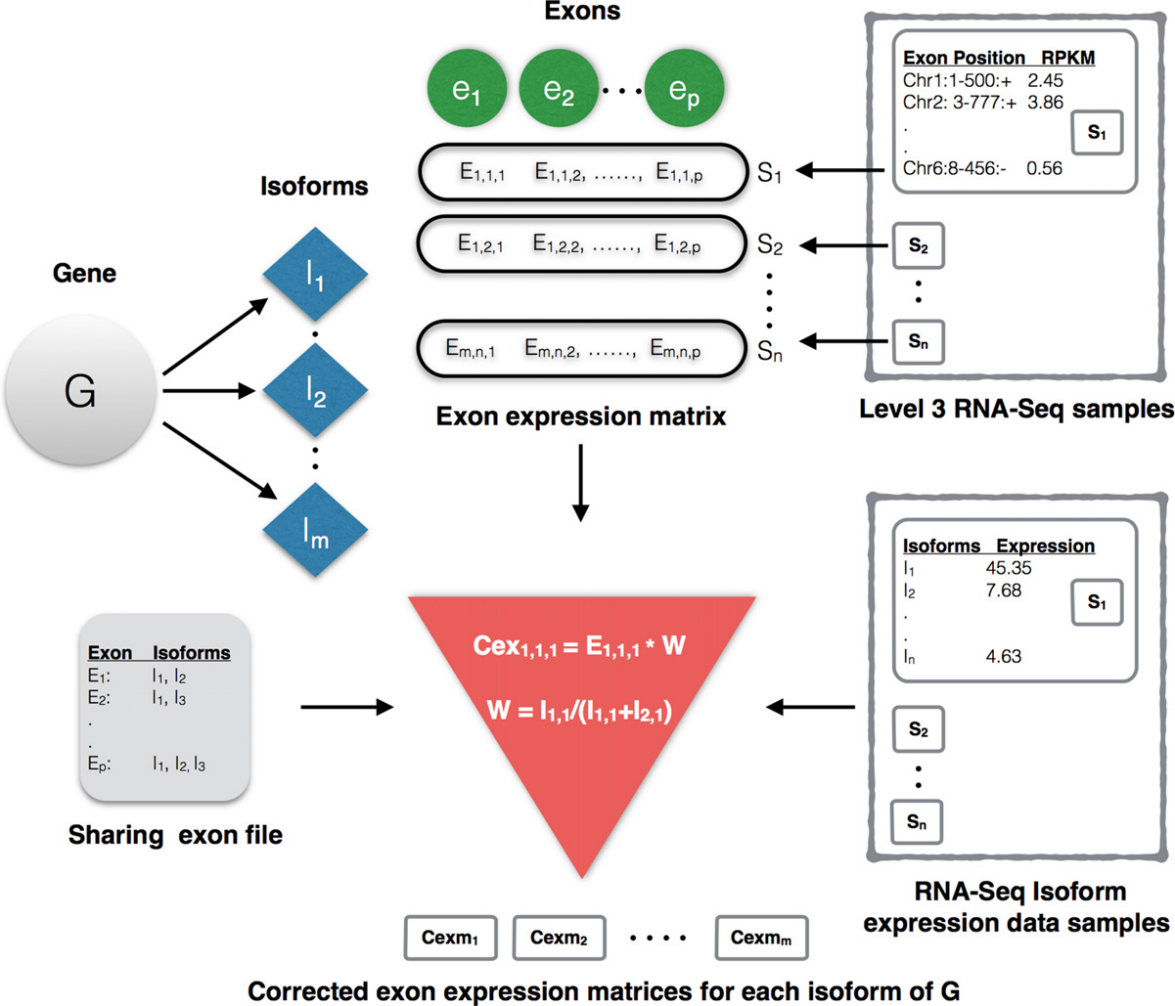
Illustration of extracting exon-expression matrix for each isofom of a gene in interest list. *G* is the gene of interest, *I* is the isoform, *e* is the exon, *S* is the sample, *m* is the number of isoforms, *p* is the number of exons, *n* is the number of samples. *E_m,n,p_*, Cex*_m,n,p_* and *W_m,n,p_* are raw expression, corrected expression and correction weight for *p*th exon of *m*th isoform in *n*th sample, respectively. *I_m,n_* is the expression of *m*th isoform in *n*th sample (from isoform expression files). It can be observed that exon *E*_1_ is shared by two isoforms *I*_1_ and *I*_2_. Thus, corrected exon-expression value of exon 1 in sample 1 for isoform 1 is computed as Cex_111_ = [*E*_111_ × {*I*_1,1_/(*I*_1,1_ + *I*_2,1_)}].

Every isoform of a gene in the interest list is represented as an exon-expression matrix (multivariate random variable) of order *p* × *n*, where *p* is the number of exons mapped to the isoform and *n* is the number of samples (RNA-Seq) as illustrated in Figure 1. Firstly a gene *G* is mapped to its isoforms and then to their corresponding exon boundaries according to the coordinates of HG-19 (UCSC genome browser) reference genome. Secondly, exon boundaries of each isoform from 1,.., *m* of gene *G* are matched to exon-positions of each level 3 RNA-Seq sample and corresponding exon-expression values are extracted. An exon is considered only if it is expressed in at least 50% of the samples, as any inference with half of the data missing (no expression) is not reliable. Considering sequencing errors an error margin of ±5 nt positions is allowed in mapping exon boundaries. The error margin of ±5 nt is a reasonable tradeoff between the acceptable sequencing errors and the smallest human exon of 15 nt (28) and can avoid imprecise exon mappings. Thus, each isoform is represented as an expression matrix with exons and samples as columns and rows respectively. However, it is well established that a significant fraction of mammalian genes overlap and share common exons. In the light of this fact it is not reasonable to assign same expression value to an exon for all its instances that are shared by multiple isoforms/genes. This makes it difficult to distinguish isoforms that share a significant number of exons or overlapping genes and is not accounted by previous studies (17,18). Moreover, isoform expression is tissue- and condition-specific i.e. isoforms of a gene express differentially in different tissues and conditions. Assigning the same expression value to all the instances of an exon will result in farcical imputations. For example, B-cell lymphoma-extra, Bcl-x, a very well studied cancer associated gene, has two isoforms Bcl-xS (short) and Bcl-xL (long). The two isoforms differ only by one exon but with totally distinct expressions and functions. Any inferences using uniform exon-expression values for both the isoforms will be inaccurate. This problem is addressed by normalizing the expression value of each instance by relative abundance of the corresponding isoform in a specific sample. Firstly, all known HG-19 isoforms are scanned for shared exon boundaries and summarized to a sharing exon file with each row representing an exon and its isoform instances as shown in Figure 1. Corrected exon-expression value for each isoform is computed as follows:

(1) \begin{equation*} {\rm Cex}_{m,n,p} = E_{m,n,p} \times W_{m,n,p} \end{equation*}

(2) \begin{equation*} W_{m,n,p} = \frac{{I_{mn} }}{{\sum\nolimits_{i = 1}^K {I_i } }} \end{equation*}

where Cex*_m,n,p_*, *E_m,n,p_* and *W_m,n,p_* are corrected expression, raw expression and correction weight of *p*th exon in *n*th sample for *m*th isoform, *I_mn_* is the expression of *m*th isoform in *n*th sample and *K* is the number of isoforms sharing a common exon *p*. This normalizes every instance of an exon with the relative abundance of the corresponding isoform and sample. For example, from Figure 1 it can be observed that exon *E*_1_ is shared by two isoforms *I*_1_ and *I*_2_. Thus, corrected exon-expression value of exon 1 in sample 1 for isoform 1 is computed as Cex_111_ = [*E*_111_ × {*I*_1,1_/(*I*_1,1_ + *I*_2,1_)}]. Sample wise exon-level expressions and isoform expressions are downloaded from TCGA data portal.

### Constructing isoform co-expression networks using large dimensional trace (LDT)

Isoform-specific co-expression networks are constructed by identifying pairwise dependencies between the isoforms of different genes, using exon-level RNA-Seq data. Previous studies have used classical statistical methods, which are designed under the assumption that the number of exons per gene (dimensions) is small while the sample size is sufficiently large (17,18). However, when both number of exons per gene and sample size are large with comparable magnitude, the classical methods are no longer effective. To handle such situations an LDT method is employed in this study. The asymptotic results of LDT are derived using large dimensional theory, where dimensions of data are significantly large together with the sample size. The proposed method abstracts expressions of genes as multivariate random variables with different number of dimensions (exons). Consider two isoforms/genes ***X***^(**1**)^ and ***X***^(**2**)^ with *p* and *q* number of exons respectively. Exon-level expressions of the sample are represented as $\left[ {x_1^{(1)} ,..,x_p^{(1)} } \right]^T \;{\rm and}\;\left[ {x_1^{(2)} ,..,x_q^{(2)} } \right]^T$ respectively. $x_i^{(1)} \;{\rm and}\;x_i^{(2)}$ correspond to the expression of the *i*th exon in ***X***^(**1**)^ and ***X***^(**2**)^ and the sample size is *n*. Suppose that the exon-expression matrix ${\bf X} = \left[ {\begin{array}{*{20}c} {{\bf X}^{(1)} } \\ {{\bf X}^{(2)} } \\\end{array}} \right]$ follows a (*p* + *q*)-dimensional normal distribution *N*(***μ*,*Σ***), where ***μ*** is the mean vector and ***Σ*** is the population covariance matrix of ***X***.

(3) \begin{eqnarray*} &&{\boldsymbol \mu } = E({\boldsymbol X}) = \left( {\begin{array}{*{20}c} {{\boldsymbol \mu } _1 } \\ {{\boldsymbol \mu } _2 } \\ \end{array}} \right)\;{\rm and}\; \nonumber \\ &&{{\boldsymbol \varSigma}} = E({\boldsymbol X} - E({\boldsymbol X}))({\boldsymbol X} - E({\bf X}))^{\rm T} = \left( {\begin{array}{*{20}c} {{\boldsymbol \varSigma _{11}} } & {{\boldsymbol \varSigma _{12}} } \\ {{\boldsymbol \varSigma _{21}} } & {{\boldsymbol \varSigma _{22}} } \\ \end{array}} \right), \end{eqnarray*}

where ***Σ***_11_ and ***Σ***_22_ are the variance matrices of ***X***^(**1**)^ and ***X***^(**2**)^ respectively, and ***Σ***_12_ is the covariance matrix of ***X***^(**1**)^ and ***X***^(**2**)^, ***Σ***_21_ being the transpose form of ***Σ***_12_.

In particular, ***Σ***_12_ = 0 identifies a zero correlation and independence between the two multivariate random variables, ***X***^(**1**)^ and ***X***^(**2**)^. Accordingly, the null hypothesis of two independent isoforms (sets of variables) is represented as follows:

(4) \begin{equation*} H_0 :{\boldsymbol \varSigma _{12}} = {\bf 0}\quad {\rm versus}\quad H_1 :{\boldsymbol \varSigma _{12}} \ne {\bf 0}. \end{equation*}

The unbiased estimators of ***Σ****_ij_* are

(5) \begin{eqnarray*} && \hat {\boldsymbol \varSigma _{ij}} = \frac{1}{{n - 1}}\sum\nolimits_{k = 1}^n {\left( {x_k^{(i)} - \bar x^{(i)} } \right)\left( {x_k^{(j)} - \bar x^{(j)} } \right)^{{\rm T}} } , \nonumber \\ && \bar x^{(i)} = \frac{1}{n}\sum\nolimits_{k = 1}^n {x_k^{(i)} } \;{\rm for}\;i {\rm\, and\,}j = 1\;{\rm and}\;2. \end{eqnarray*}

To test the hypothesis *H*_0_, we use the LDT statistic defined as follows:

(6) \begin{equation*} L_n = {\rm tr}({\boldsymbol A}_{{21}} {\boldsymbol A}_{{11}}^{{\boldsymbol - 1}} {\boldsymbol A}_{{12}} {\boldsymbol A}_{{22}}^{ {\boldsymbol - 1}} ), \end{equation*}

(7) \begin{equation*} {\boldsymbol A}_{{ij}} = (n - 1)\hat {\boldsymbol \varSigma} _{{ij}} , \end{equation*}

where *tr* denotes the trace of a matrix. The elements on the main diagonal of $({\boldsymbol A_{21} {\boldsymbol A}_{11}^{ - 1} {\boldsymbol A}_{12} {\boldsymbol A}_{22}^{ - 1}} )$ comprehend the essential information of correlation between the exons of respective isoforms/genes. Thus, the sum of these diagonal elements, defined as trace, quantifies the degree of dependency among isoforms. Under the null hypothesis, the statistic *L_n_* converges to a normal distribution and is close to zero. A co-expression edge is drawn between any two isoforms/genes based on accepting or rejecting the null hypothesis by comparing the observed value of test statistic, *T* to the critical value *Z* at significance level *α*. If the null hypothesis is rejected, an edge is inferred connecting corresponding isoforms. The critical value for testing the hypothesis is computed by deriving an asymptotic distribution of the statistic (29). As $p,q \to \propto$ and $n \to \propto$, the asymptotic distribution of *L_n_* is as follows:

(8) \begin{equation*} T = V^{ - \frac{1}{2}} (L_n - E) \to N(0,1) \end{equation*}

(9) \begin{equation*} V = \frac{{2pq(n - 1 - p)(n - 1 - q)}}{{(n - 1)^4 }}\;{\rm and}\;E = q \times \frac{p}{{n - 1}}, \end{equation*}

where *V* is the variance and *E* is the expected value of *L_n_*. A co-expression edge is placed if *T* > *Z_α_* at significance level *α*. The critical value *Z_α_* is the *α*th upper quantile of standard normal distribution. Intuitively, the edges can be weighted according to the *P*-value of the corresponding test statistic *T*. Compared to traditional criteria in multivariate analysis for testing the independence hypothesis, the advantage of the LDT criterion is that it can handle large datasets with large dimensions *p* and *q*, provided that the ratios *p*/*n* and *q*/*n* are close to 1.

In contrast, the CCA criterion is based on standard consistent estimate of population CCA, provided that the dimensions *p* and *q* are small enough compared to sample size (low-dimensional assumption). When the ratios of dimension to sample size *p*/*n* and *q*/*n* are not small enough (e.g. *p* = *q* = 20, *n* = 50), from recent high-dimensinonal statistic literature, we knew that standard estimation is not consistent. Therefore, test procedure based on CCA is not reliable. Experiments in the results section clearly show that SpliceNet significantly outperforms CCA.

### Inferring differential cancer co-expression networks

The method described in the previous section can essentially infer isoform-specific co-expression networks from cancer and normal samples (RNA-Seq data) respectively. Nevertheless, to systematically understand the cause, prognosis and to identify confident therapeutic targets it is very important to distinguish cancer and normal samples. Differentially expressed genes are often identified as disease causing/target genes. The limitation of discounting relationships among genes in such studies advocates the need of new approaches. This study goes beyond differentially expressed genes and theorizes genes as networks to thoroughly comprehend a disease by inferring differential cancer co-expression networks.

**Figure 2.**
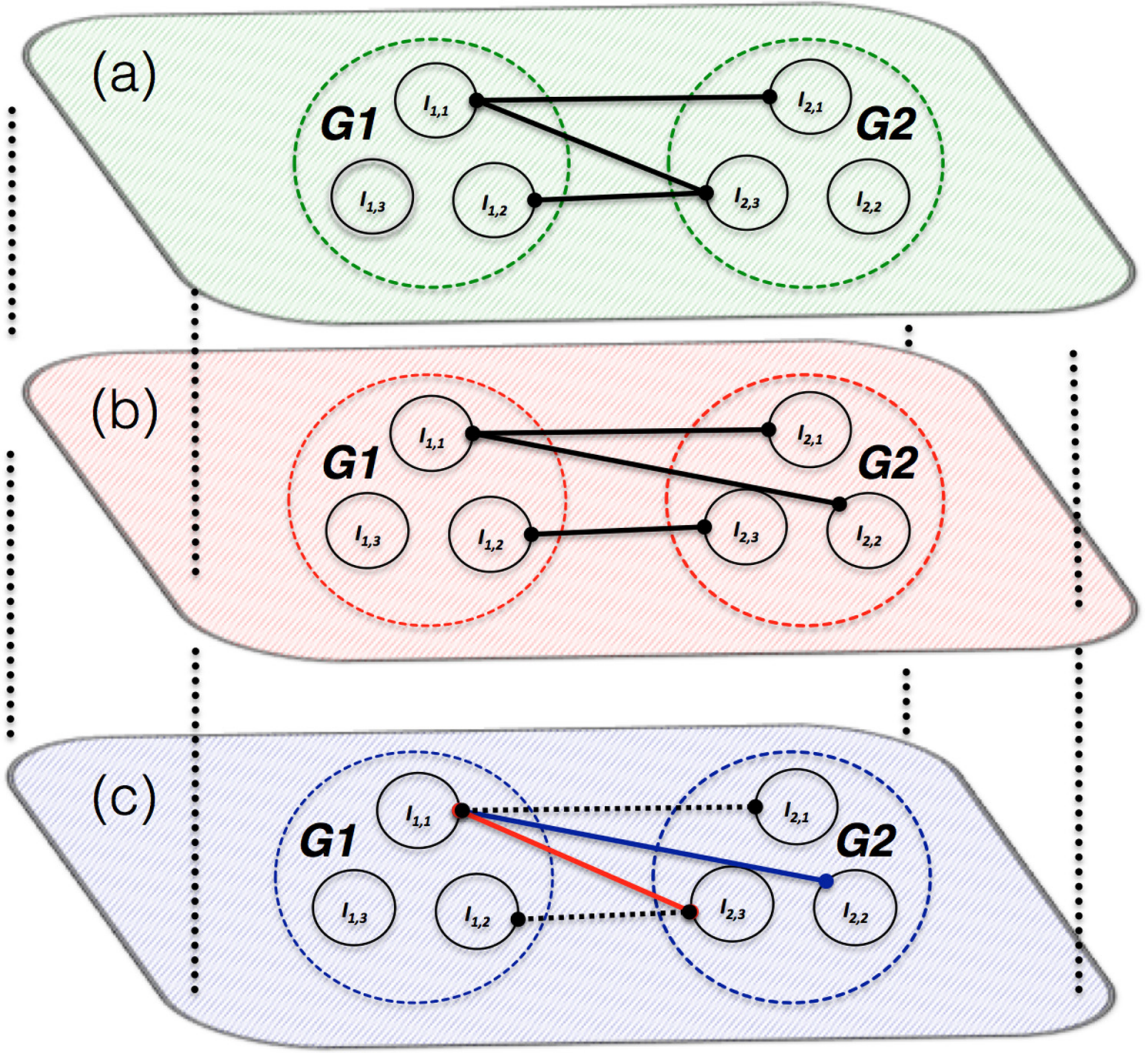
Illustration of inferring differential cancer co-expression network: isoform-specific co-expression network inferred from (**a**) Normal samples, (**b**) cancer samples and (**c**) differential cancer network. Solid lines in red and blue are the edges lost and gained in cancer samples respectively when compared to normal samples. Dotted lines are the removed common edges.

A differential cancer co-expression network is defined as a network with co-expression edges that are either observed only in cancer or in normal samples. Firstly, two independent co-expression networks are inferred using the proposed methods from tumor-matched and normal-matched RNA-Seq samples respectively. Then, a graph comparison operation is performed to remove all common edges. The remainder, differential co-expression edges can be ranked based on the corresponding *P*-values. According to Figure 2a, in normal samples isoform *I*_1,1_ of gene *G*_1_ is co-expressed with isoforms *I*_2,1_ and *I*_2,3_ of gene *G2*, and *I*_1,2_ of *G*_1_ with *I*_2,3_ of *G2*. On the other hand, in cancer samples (Figure 2b), *I*_1,1_ of *G*_1_ is co-expressed with *I*_2,1_ and *I*_2,2_ of *G2*, and *I*_1,2_ of *G*_1_ with *I*_2,3_ of *G2*. A differential cancer co-expression network in constructed by removing common edges, *I*_1,1_ *– I*_2,1_ and *I*_1,2_ *– I*_2,3_. Thus the resultant differential network (Figure 2c) has two edges, *I*_1,1_ *– I*_2,2_ (blue) and *I*_1,1_ *– I*_2,3_ (red).

## RESULTS

**Figure 3.**
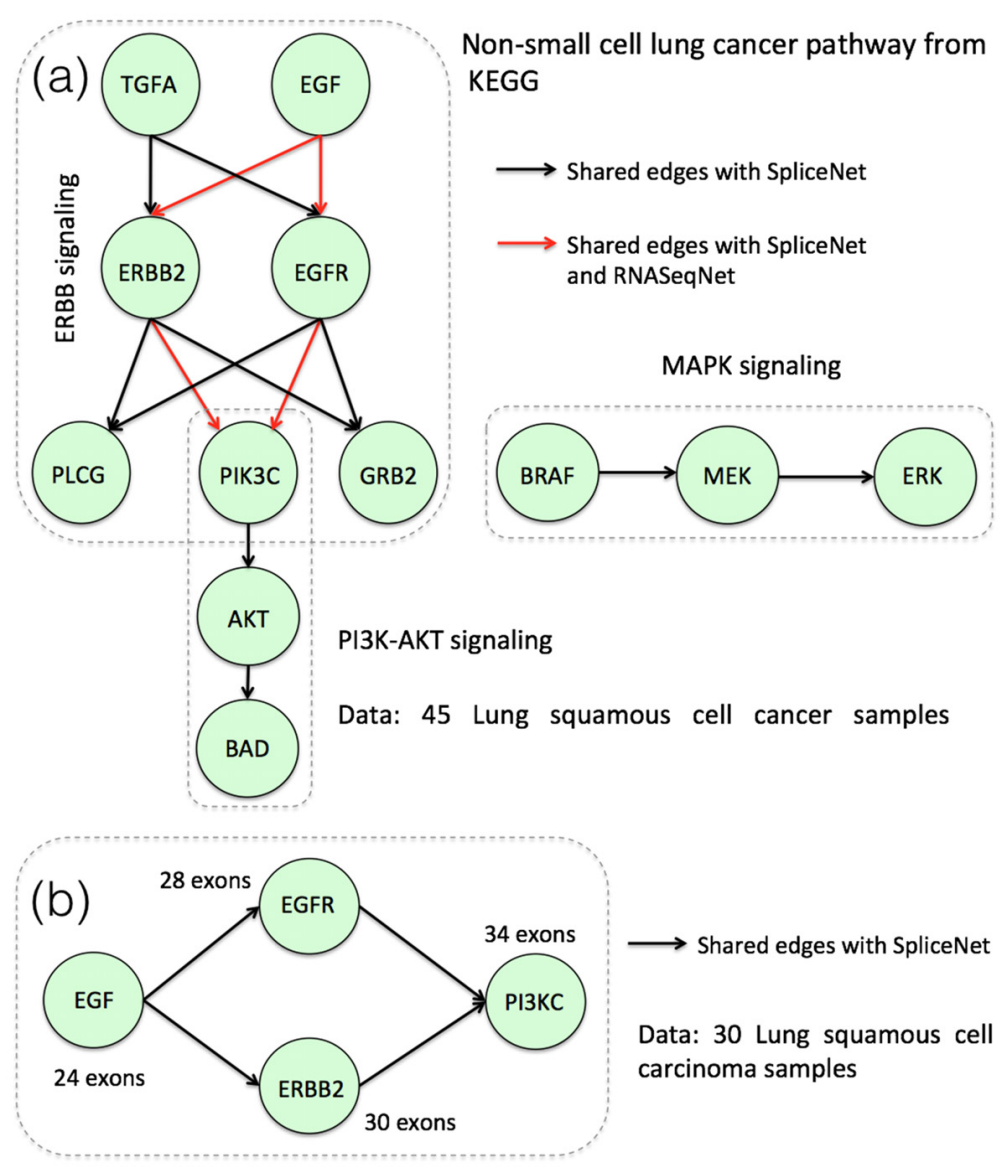
(**a**) Inferred non-small cell lung cancer pathway using the SpliceNet and RNASeqNet. (**b**) Re-inferred ERBB2 signaling pathway, but with a reduced sample size.

**Figure 4.**
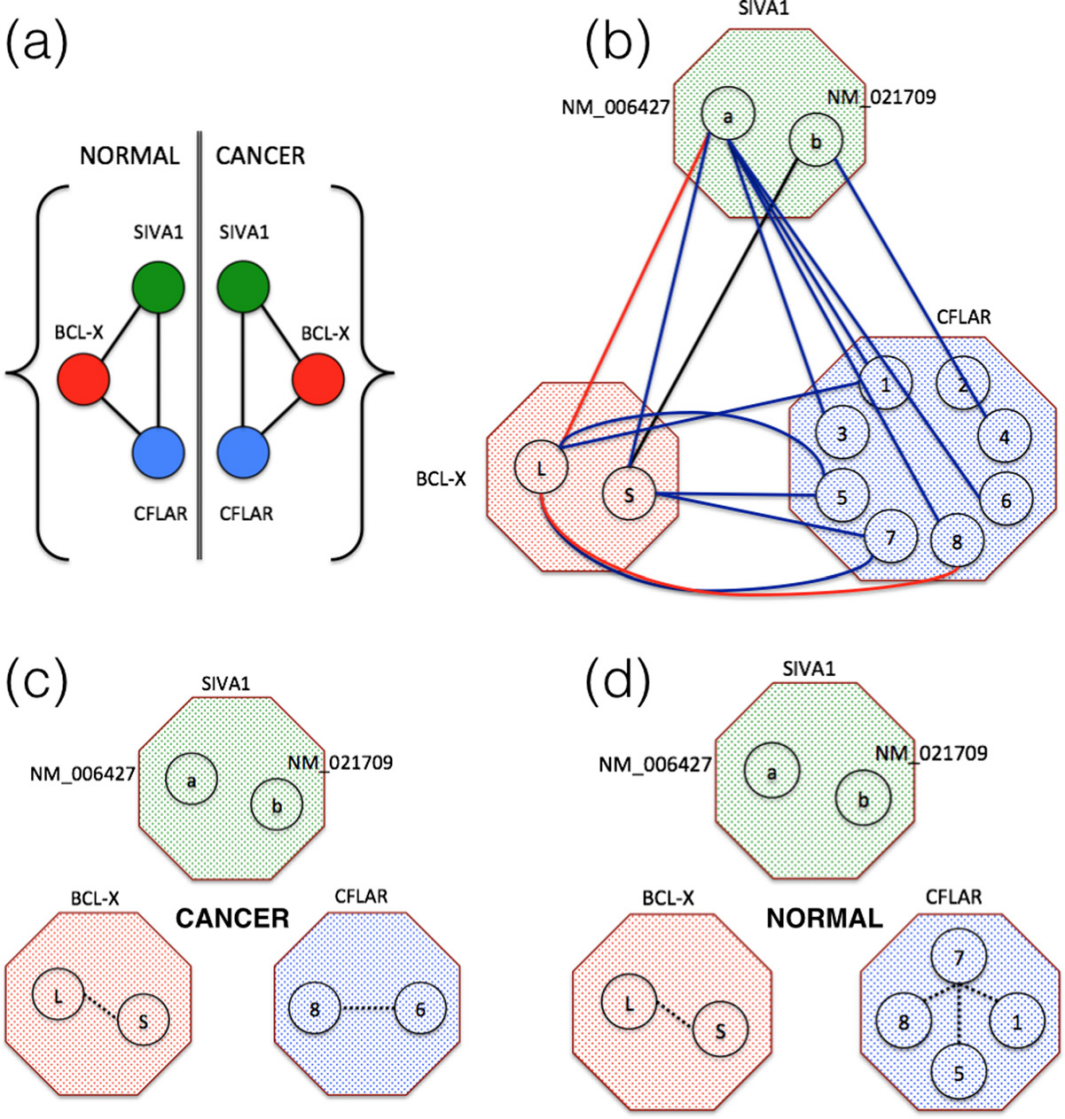
(**a**) Gene level dependencies from normal and cancer samples. (**b**) Differential network with isoform resolution, of the same. Shown in black are the dependencies observed in both cancer and normal samples, in red are the dependencies lost and in blue are the dependencies gained in cancer samples when compared to normal samples. (**c**) Dependencies inferred by RNASeqNet from cancer and (**d**) normal samples. CFLAR isoforms: 1-NM_001202519, 2-NM_001202515, 3-NM_001127183, 4-NM_001127184, 5-NM_003879, 6-NM_001202518, 7-NM_001202516 and 8-NM_001202517.

**Figure 5.**
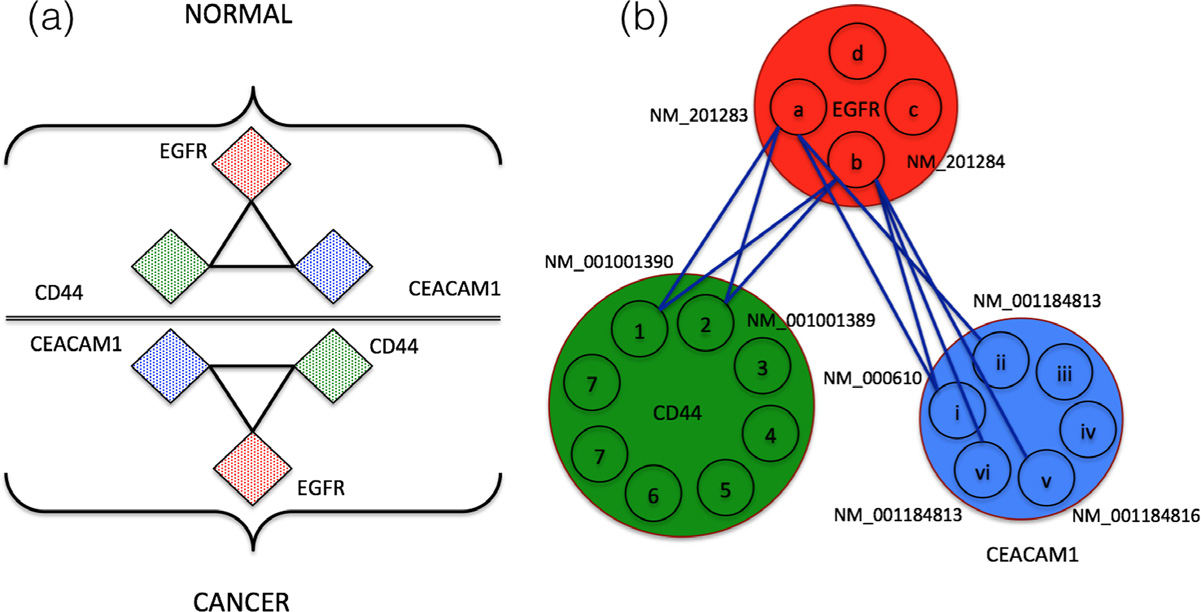
(**a**) Gene level dependencies from normal and cancer samples. (**b**) Differential edges of the same.

The key merit of SpliceNet is in handling large dimensional data, where the number of exons per gene is large and comparable to sample size i.e. when the ratio of number of exons per gene to sample size is large. Firstly, to thoroughly evaluate the performance and stability of SpliceNet, simulations are performed by varying number of exons (dimensions) and samples. The performance of existing R package, RNASeqNet is also evaluated on the same data. The results summarized in Table 1 demonstrate the competence of SpliceNet in abstracting dependencies from exon-expression (high-dimensional) data. Secondly, SpliceNet and RNASeqNet are evaluated on cancer-specific ERBB2 and MAPK signaling pathways from KEGG database with different number of samples. The results summarized in Figure 3 evince the merit of SpliceNet over RNASeqNet in handling low sample datasets. Further, to appreciate the insights of differential cancer networks and their applications, a detailed work out of SpliceNet on Bcl-x and EGFR centered network is illustrated (Figures 4 and 5). Differential edges inferred by SpliceNet converged to cancer-specific splice variants reported in literature. Finally, to demonstrate the practical pertinence, performance of SpliceNet is also evaluated on real RNA-Seq data from three different tissues viz. lung, kidney and liver, alongside RNASeqNet. The *F*-scores reported in Table 4 demonstrate a significantly enhanced performance of SpliceNet over RNASeqNet.

**Table 1.** *F*-scores of SpliceNet and RNASeqNet on simulated data with varying number of exons (dimensions), sample size and *c*_0_ (inverse noise level) values

*c*_0_	Gene pair		Number of samples							
	G 1	G 2	25		50		75		100	
			RNASeqNet	SpliceNet	RNASeqNet	SpliceNet	RNASeqNet	SpliceNet	RNASeqNet	SpliceNet
0.2	5	5	0.132	0.684	0.258	0.713	0.393	0.744	0.525	0.769
	20	20	NaN^a^	0.671	0.174	0.696	0.319	0.731	0.491	0.763
	20	5	0.575^b^	0.668	0.116	0.682	0.177	0.697	0.272	0.715
0.4	5	5	0.416	0.748	0.657	0.791	0.677	0.795	0.677	0.794
	20	20	NaN^a^	0.685	0.582	0.786	0.675	0.793	0.678	0.795
	20	5	0.572^b^	0.676	0.420	0.749	0.620	0.786	0.665	0.793
0.6	5	5	0.652	0.782	0.675	0.793	0.677	0.795	0.68	0.795
	20	20	NaN^a^	0.702	0.681	0.795	0.678	0.790	0.679	0.793
	20	5	0.580	0.694	0.654	0.791	0.676	0.796	0.679	0.794

^a^Total number of exons is greater than sample size.

^b^Performance drop of RNASeqNet.

**Table 2.** Performance of RNASeqNet on simulated data II showing the relative importance of dimensions to sample size ratio over sample size

G1	G2	*F*-score/number of samples			
5	5	0.55/10	0.09/20	0.14/30	0.20/40
10	10	0.57/20	0.11/30	0.17/40	0.23/50
15	15	0.57/30	0.14/40	0.19/50	0.27/60

**Table 3.** Correlation between different tissue types with respect to overall gene expressions from Human BodyMap 2.0

Tissue	Kidney	Liver	Lung
Adipose	0.826	0.563	0.235
Blood	0.267	0.240	0.502
Brain	0.764	0.530	0.173
Breast	0.834	0.579	0.328
Colon	0.897	0.581	0.251
Heart	0.789	0.570	0.121
Kidney	1.000	0.624	0.180
Liver	0.624	1.000	0.216
Lung	0.180	0.216	1.000
Lymph	0.047	0.136	0.905
Ovary	0.518	0.463	0.521
Prostate	0.525	0.409	0.353
Skeletal muscle	0.808	0.643	0.117
Testes	0.788	0.495	0.171
Thyroid	0.679	0.527	0.222

**Table 4.** Gene level evaluation of SpliceNet and RNASeqNet on real RNA-Seq samples from lung, kidney and liver tissues

Tissue	SpliceNet	RNASeqNet
Lung	0.76	0.64
Liver	0.69	0.62
Kidney	0.73	0.66

### Simulation study

Simulations are performed by varying number of exons per gene (dimensions) and samples to analyze the influence of the same on the performance of SpliceNet. For gene pair G1–G2, number of exons are set to 5-5 (low), 20-20 (high) and 20-5 (high-low), and number of sample to 25, 50, 75 and 100 i.e. in total there are 12 experimental setups. For every setup, 100 000 replications are performed at 5% significance level i.e. a dependency is considered statistically significant if the *P*-value is ≤0.05. For independent gene pair (no co-expression), random sample ***Z*** = (***Z_1_ Z_2_***)^T^ is drawn from population following multivariate normal distribution ***N***(0,*I*) of sample size *n*, where ${\boldsymbol Z}_i = (z_{i1} , \ldots ,z_{ip_i } )^{{\rm T}}$, *i* = 1,2 and *p* is the number of exons. For dependent gene pairs (co-expressed), sample ***X*** = (***X_1_ X***_***2***_)^T^ is drawn such that

(10) \begin{eqnarray*} &&{\boldsymbol X}_i = {\boldsymbol Z}_i + c_0 {\boldsymbol Z}_m^{p_i } ,i = 1,2;\nonumber\\&&{\boldsymbol Z}_m^{p_i } = \left\{ {\begin{array}{*{20}c} {{\boldsymbol Z}_1^{p_i } = (z_{11} , \ldots ,z_{1p_i } )^{\rm T} ,} & {p_1 \ge p_2 ,} \\ {{\boldsymbol Z}_2^{p_i } = (z_{21} , \ldots ,z_{2p_i } )^{\rm T} ,} & {p_1 {<} p_2 .} \\ \end{array}} \right. \end{eqnarray*}

where *c*_0_ is a constant that is inversely proportional to the distance between null and alternative hypothesis. The performance and stability of SpliceNet is demonstrated by simulating each experimental setup with three different *c_0_* values, *0.2*, *0.4* and *0.6*. A measure of accuracy, *F*-score (30) is reported for each experimental setting in Table 1. The *F*-score measures the trade-off between precision *p* and recall *r*.

(11) \begin{equation*} F = 2\times \frac{{p \times r}}{{p + r}} \end{equation*}

\begin{eqnarray*}
&&p = \frac{{true\;positives}}{{true\;positives + false\;positives}}; \nonumber \\
&&r = \frac{{true\;positives}}{{true\;positives + false\;negatives}}
\end{eqnarray*}

From Table 1, firstly it can be observed that the performance of RNASeqNet significantly dropped with the increase in noise (inversely proportional to *c_0_*). In contrast the performance of SpliceNet is extremely stable between *c_0_* values 0.6 and 0.4, and adequately stable between 0.4 and 0.2. The overall performance drop of SpliceNet is <10%, verifying the stability of SpliceNet. Secondly, number of exons and sample size are also found to influence the performance of respective methods. A general trend of increasing performance is observed as the sample size increases from 25 to 100 for both RNASeqNet and SpliceNet. However, the performance of SpliceNet is quite significant when compared to RNASeqNet even with smaller sample size and stabilizes quickly (at sample size 50 in the current experimental setup). This demonstrates the suitability of SpliceNet even to smaller datasets, which is a major bottleneck for the current methods. Efficiently handling smaller sample size is one of the prime requirements of any analytical tool in biological domain, as it is not always practical to have large number of samples of a specific cancer/disease/condition, small number of available tumor and normal matched RNA-Seq samples support this argument. The *F*-scores of SpliceNet on different exon combinations 5-5 (low), 20-20 (high) and 20-5 (high-low) are quite comparable, with maximum at 5-5 followed by 20-20 and 20-5. This suggests the merit of SpliceNet in handling genes with both small and large number of exons. It is important to note that SpliceNet has effectively handled high dimensional cases (20-20), especially when the total number of exons (40) is greater than the sample size (25). In contrast, RNASeqNet failed to make any inferences when total number of exons is greater than sample size (marked by superscript a in Table 1). In addition, the performance of RNASeqNet on 20-5 exon combination dropped sharply (marked by superscript b in Table 1) and was shadowed by a slow increase (at *c_0_* values 0.2 and 0.4) as the sample size increased from 25 to 100. This phenomenon suggests the influence of dimensions to sample size ratio than just the sample size on the performance of CCA based RNASeqNet. In contrast, an increasing trend of performance is observed for other combinations (5-5 and 20-20). It is speculated that a square matrix structure, when the sample size (25) is exactly equal to the total number of exons (20 + 5) is relatively important than sample size for RNASeqNet. To validate this speculation, RNASeqNet is evaluated on a second simulated dataset representing the conditions described above with medium noise level (*c_0_* = 0.4), and the results are summarized in Table 2.

The performance of RNASeqNet (Table 2) dropped sharply first and then increased slowly, as the sample size increased. This supports the suspicion on the relative importance of dimensions to sample size ratio (square matrix structure) over sample size. However, it is not valid at low noise level (*c_0_* = 0.6), raising consistency concerns on the performance of CCA. Over all, it is evident from Table 1 that SpliceNet outperformed RNASeqNet in all the experimental setups. Precision of SpliceNet is slightly better than recall when the sample size is small. However, they are almost equivalent when the sample size is moderated to large (see the Supplementary Data). The stability of SpliceNet at different noise levels and consistency with varying exon to sample size ratios makes it best suitable for practical applications when compared to RNASeqNet.

### Evaluation on cancer-specific ERBB2 and MAPK signaling pathways

To draw a parallel, SpliceNet is evaluated on the same non-small cell lung cancer-specific pathway used by RNASeqNet (18). Cancer-specific ERBB2 and MAPK signaling pathways are downloaded from KEGG database. Firstly, a total of 45 LUSC matched samples are used to infer the edges and the results are summarized in Figure 3a. Shown in black are the true edges that are also inferred by SpliceNet and shown in red are the true edges that are inferred by both SpliceNet and RNASeqNet. It can be observed from Figure 3a that RNASeqNet inferred only four edges using 45 samples in contrast to what is observed using 225 samples (18). On the other hand, SpliceNet recovered all the true edges. Next, the sub network that is inferred by RNASeqNet with 45 samples (red edges in Figure 3a) is re-inferred, but with a reduced sample size 30 and the results are shown in Figure 3b. As the total number of exons (dimensions) of any two genes is greater than the sample size (30), RNASeqNet failed to infer any edge (see the Supplementary Data). In contrast, the performance of SpliceNet is least affected. Over all, Figure 3a and b evince the merit of SpliceNet over RNASeqNet in handling high exon to sample size ratio (smaller sample size) datasets.

### Isoform-specific differential cancer networks from non-small cell lung adenocarcinoma (LUAD) samples

To comprehend the advantages and the applications of isoform-specific Differential Cancer Networks, a detailed work out of SpliceNet on Bcl-x and EGFR centered network is demonstrated here. Bcl-x gene is well established to be involved in majority of non-small cell lung cancers (31). It has two splice variants Bcl-xL and Bcl-xS with anti-apoptotic and pro-apoptotic functions respectively (32). Dependence of Bcl-x on SIVA1 and CFLAR genes are investigated in both cancer and normal samples and respective networks are shown in Figure 4. Proteins encoded by SIVA1 and CFLAR play an important role in apoptosis cycle (tumorigenesis) and are reported to be interacting with Bcl-x (33,34). It can be observed from Figure 4a that there is no difference between the networks derived from cancer and normal samples, and is difficult to explain tumorigenesis. Hence it is imperative to investigate isoform interactions to decode the underlying tumorigenic molecular interactions. SpliceNet offers intuitive conclusions in understanding the role of molecular interactions in various biological phenomena, here LUAD. Figure 4a shows the differential network with isoform resolution, of the same. The differential edge (red) between Bcl-xL and SIVA1-NM_006427 hints at role of Bcl-xL in cancer. The role of Bcl-xL can also be inferred by relative isoform expressions. However, the mechanism still remains an unsolved puzzle. The inferred differential edge suggests a possible loss of dependency (interaction) between Bcl-xL and SIVA1-NM_006427, which is in agreement with literature. SIVA1 binds to Bcl-xL to inhibit its anti-apoptotic function (33). Thus in cancer samples the corresponding dependency is lost. The dependency here indicates co-expression including molecular interaction. CFLAR can act as a critical link between cell death and survival pathways in mammalian cells. Both the isoforms of Bcl-x have differential edges to the isoforms 5 and 7 of CFLAR. Additionally, Bcl-xL also has differential edges to CFLAR 1 and 8 indicating their relative importance in lung tumorigenesis. However, CFLAR isoform functional differences are still unclear and such inferences needs to be experimentally validated. On the other hand, RNASeqNet recovered only intra gene dependencies from both cancer and normal samples (Figure 4c and 4d). Only a possible role of CFLAR isoforms in cancer can be inferred from this, which can also be concluded from a simple differential expression study. Detailed isoform dependencies in normal and cancer samples respectively are given in the Supplementary Data

Another small network including EGFR (four isoforms) and two other well-established cancer related genes viz. CD44 (35), eight isoforms and CEACAM1 (36), 6 isoforms, is studied at both gene and isoform levels. The results are summarized in Figure 5. Both CD44 and CEACAM1 are reported to have interactions with EGFR in STRING database (37) with experimental evidence. It can be observed from Figure 5a that there is no difference in dependencies inferred from normal samples and cancer samples i.e. the genes EGFR, CD44 and CEACAM1 are co-expressed in both cancer and normal samples and does not give any insights into respective tumorigenic mechanisms. On the other hand, isoform-specific dependencies (Figure 5b) revealed cancer associated isoforms of EGFR. Out of the four isoforms of EGFR, NM_201283 and NM_201284 have edges only in cancer samples (Figure 5b and Supplementary Data) suggesting their importance in tumorigenesis when compared to other two isoforms. EGFR variant 3, NM_201283 is reported to be strongly associated to lung cancer by several studies (38–40). Exploring NM_201283's differential edges, critical isoforms of other genes can also be inferred. CD44 variant, NM_001001390 and CEACAM1 variant, NM_000610 are found to be linked to NM_201283 of EGFR, in cancer samples and are also in reported to be critical in non-small cell lung cancers (41,42). The same are also differentially linked to NM_201284 of EGFR in agreement with the earlier observation (NM_201283 and NM_201284 have edges only in cancer samples). Detailed isoform-specific differential dependencies of EGFR centered network are given in the Supplementary Data.

### Gene level evaluation of inferred co-expressions from RNA-Seq data

To demonstrate the practical applicability, SpliceNet is also evaluated on real RNA-Seq data from three different tissues viz., lung, kidney and liver. Only normal-matched RNA-Seq samples are used for the following evaluation. A total of 49 lung adenocarcinoma (LUAD), 50 LIHC and 72 KIRC samples are downloaded from TCGA data portal. Due to the lack of adequate experimental evidence for isoform co-expression networks, evaluation is performed at gene level. Firstly, tissue-specific gene lists and gene expressions are downloaded from tissue-specific gene expression and regulation, TiGER database (25), and Ensembl's Human BodyMap 2.0 (26) respectively. From the extracted tissue-specific gene lists, 100 gene pairs belonging to the same tissue are labeled as positive pairs i.e. co-expressed and another 100 gene pairs belonging to different tissues are labeled as negative pairs (no co-expression). Despite of using tissue-specific genes, a small fraction of negative gene pairs (from different tissues) may be co-expressed. This is because, the gene lists from TiGER database are not true tissue-specific genes, but significantly expressed in a specific tissue. To avoid any such correlated pairs in negative dataset, tissues for compiling the negative pairs are chosen such that the overall gene expression correlation between them is the least. This ensures the heterogeneity between tissues and there by minimizes correlated pairs in negative dataset. Comprehensive gene expressions for each tissue type are collected from Ensembl's Human BodyMap 2.0 and respective correlations are computed (Table 3). It can be observed from Table 3 that skeletal muscle, lymph and lung are least correlated with lung, liver and kidney, and thus used to compile negative datasets respectively. Accordingly, three sets of positive and negative datasets are extracted for lung, kidney and liver tissues. These labeled gene pairs are used as a benchmark to validate SpliceNet. To draw parallel, RNASeqNet is also evaluated on the same datasets. The *F*-scores reported in Table 4 evince a significantly enhanced performance of SpliceNet over RNASeqNet. Higher precision is observed for SpliceNet (see the Supplementary Data). Tissue-specific gene lists and gene expressions can be downloaded from TiGER database (25) and Ensembl's Human BodyMap 2.0 (26), respectively.

## DISCUSSION

Network inference is the first step towards understanding any complex biological phenomenon (3,43,44). The dynamic interplay of genes and their splice variants can help us to comprehend fundamental mechanisms in various biological abnormalities including cancer. Conventionally, microarrays are used to quantify gene expressions. However, it is challenging to account whole transcriptome using microarrays. Recent high-throughput RNA-Seq has made splice variant profiling practical. Recent studies demonstrated the use of RNA-Seq data in constructing gene networks. However, the merit of RNA-Seq in quantifying splicing isoforms is not explored in inferring isoform-specific networks. Moreover, previous studies are designed under the assumption that the number of dimensions is small while the sample size tends to infinity. This advocates the need of more robust methods investigating RNA-Seq data.

This study demonstrates a novel method to infer isoform-specific co-expression networks from exon-level RNA-Seq data using LDT. The proposed method, SpliceNet abstracts expressions of genes as multivariate random variables with different number of dimensions (exons) and tests the corresponding dependencies by approximating an empirical distribution. Isoform-specific exon expressions are computed from sample-wise isoform expression data, which was estimated by TCGA project team using RSEM algorithm (45). However, RSEM estimates may not be always accurate. In simulation study, existing method RNASeqNet (based on CCA) failed to make any inferences when total number of exons per gene (dimensions) is greater than sample size. In contrast, SpliceNet performed well suggesting its merit in handling genes/isoforms with both small and large number of exons, especially when the total number of exons is greater than the sample size. In addition, SpliceNet has an appealing property that the edge is determined by hypothesis testing instead of a discretionary threshold. Evaluation on both simulated and real RNA-Seq data substantiates the performance of SpliceNet. Recovered edges of lung cancer-specific ERBB2 and MAPK signaling pathways, with varying number of samples demonstrate the merit of SpliceNet over RNASeqNet in handling high exon to sample size ratio (smaller sample size) datasets. This study goes beyond differentially expressed genes and infers network differences between normal and diseased samples at isoform level. Inferred differential cancer networks on well established Bcl-x and EGFR centered networks in non-small cell lung cancer concede with cancer-specific splice variants reported in literature. Differential edge between Bcl-xL and SIVA1-NM_006427 hints at role of Bcl-xL association with SIVA1 in cancer. Thus, provides a more comprehensive picture to our understanding of the disease. Differential edges of CD44 variant, NM_001001390 and CEACAM variant, NM_000610 with EGFR-NM_201283 clues their collective role in cancer and are also reported to be critical in non-small cell lung cancers. Although this study demonstrates the application of SpliceNet to cancer genomic data, it can be applied to any exon level RNA-Seq data or exon array data. Furthermore, by computing intra-genic isoform dependencies SpliceNet can also infer isoform mediated auto regulatory relationships. Networks inferred by SpliceNet are non-directional. In future, SpliceNet can be extended to infer directionality by integrating Chip-Seq data (43,44), and further enhance our understanding of the underlying molecular mechanisms.

## SUPPLEMENTARY DATA

Supplementary Data are available at NAR Online.

SUPPLEMENTARY DATA
