# Supplementary material for: SpliceNet: recovering splicing isoform-specific differential gene networks from RNA-Seq data of normal and diseased samples
Source: Nucleic Acids Res. 2014 Jul 17;42(15):e121. doi: 10.1093/nar/gku577 (PMC4150760; doi:10.1093/nar/gku577)
Supplement: SUPPLEMENTARY DATA [file supp_42_15_e121__index.html]

SpliceNet: recovering splicing isoform-specific differential gene networks from RNA-Seq data of normal and diseased samples — SUPPLEMENTARY DATA 

# SpliceNet: recovering splicing isoform-specific differential gene networks from RNA-Seq data of normal and diseased samples

## SUPPLEMENTARY DATA

**Files in this Data Supplement:**

- SUPPLEMENTARY DATA
